# Application of modified seagull optimization algorithm with archives in urban water distribution networks: Dealing with the consequences of sudden pollution load

**DOI:** 10.1016/j.heliyon.2024.e24920

**Published:** 2024-01-22

**Authors:** Qichun Wang, Mingxiang Zhang, Sama Abdolhosseinzadeh

**Affiliations:** a Chongqing Vocational Institute of Engineering, Chongging, 402260, China; b School of Architecture and Engineering, Chongqing Creation Vocational College, Yongchuan, 402160, Chongging, China; c University of Mohaghegh Ardabili, Ardabil, Iran; d College of Technical Engineering, The Islamic University, Najaf, Iraq

**Keywords:** Urban water network, Consequence management, Simulation-optimization, Modified multi-objective optimization algorithm of seagull, Archive capacity

## Abstract

This study focuses on the optimization of consequence management actions in the urban water distribution network. The EPANET simulation model is employed in combination with the multi-objective modified seagull optimization algorithm (MOMSOA) based on archives for a more efficient optimization process. Two objective functions are developed: minimizing reactive activities (cost reduction) and minimizing consumed pollution mass. The utilization of shut-off valves and hydrants for isolating the network and discharging pollution is explored. Without consequence management, 84.5 kg of pollution is consumed. With 18 reactive activities, pollution consumption was reduced to 59.8 kg. Also, to compare the proposed method with other algorithms, the interaction curve between reactive activities and the amount of pollutant mass consumed was obtained using other methods, including MOSOA, NSGA-II, MOPSO, and MOSMA. According to the obtained curve, the proposed method performed better in reducing the mass of consumed pollution. Extracting optimal activities using MOMSOA and a maximum of 18 activities takes about 80 min. The MOMSOA with archive technique significantly shortens this time for real-time consequence management. The proposed approach demonstrates that increasing the archive population decreases the extraction time of interaction curves between objectives by up to 60 %. A small archive capacity slightly increases the time required to extract optimal activities due to searching for similar solutions. However, utilizing the archive capacity enables real-time optimization and consequence management in the network.

## 1 Introduction

The infrastructure of any country is considered an important platform for its life, growth, and dynamism. Hence, one of the important issues is to protect the infrastructure and provide the possibility of their suitable usage in the event of a crisis. One of a city's most crucial arteries is the water supply network [1]. It is also very important to control the quality of drinking water and maintain the health of citizens [2]. Therefore, maintaining the security of water supply facilities and taking necessary measures to prepare for critical situations is one of the main concerns of security officials in any country [3]. So far, several types of research have been done in the field of securing urban water networks after the occurrence of pollution [4]. some of the most important aspects of research are determining the optimal location of distribution network monitoring stations, determining the location of pollution occurrence, and how to manage its consequences [5]. Managing the consequences of the established crisis and making the best decision to prevent the spread of pollution and its evacuation from the network is a very important issue that can be a very suitable alternative to disabling the entire network (as a very conservative solution) [6].

The US EPA (U.S. Environmental Protection Agency) (2004) has provided recommendations to minimize the risks caused by the threats that happened by pollution in the urban water network through a protocol [7]. The general recommendations of this protocol after detecting the pollution and its place of occurrence include (a) proclaiming the danger and informing the public, (b) isolating the contaminated area in the network, (c) draining the pollution from the network and (d) a combination It is from the previous three [8]. To restore the network and reuse it, optimal placement of monitoring stations and identification of pollutant source characteristics should be in such a way that implementation solutions need the least cost and time, and also the least number of people are affected by the pollution [9]. In this study, it is assumed that the presence of pollution has been detected and confirmed, and the characteristics of the source of pollution in the network have been identified [10]. Therefore, in the following, the necessary executive measures must be determined to manage the consequences of pollution [11]. These measures include isolating the pollution using shut-off valves and emptying the pollution using hydrants in the network in the shortest possible time [12]. The purpose of this research is to extract the optimal exploitation activities (consequence management) in the water distribution system for cities with the approach of reducing the optimization time [13]. Based on this, the use of a multi-objective modified seagull optimization algorithm based on archives has been considered in this research [14].

### 1.1 Related works

Consequence management is considered an effective tool to protect people's health, restore basic government services, and provide emergency assistance to governments, companies, institutions, and individuals after a pollution incident [15]. The meaning of pollution in this research is any biological, chemical, and radiological pollution that has adverse effects on public health and its surroundings [16]. The history of conducting studies in the field of consequence management in urban water distribution networks goes back about a decade. Therefore, the studies conducted in this field are very new and limited.

M Qiu et al. [17] suggested a structure for creating a real-time disinfecting strategy for a pollution incident in water distribution systems by first dividing a WDS into several district-metered areas (DMAs), then creating a solution region for each DMA, and then putting the solution region developed into use to create an efficient decontamination plan. They have used this paradigm for three contamination occurrences. The findings demonstrate that the suggested framework may be used to (1) dramatically shorten reaction times, (2) enhance the standard of decontamination plans, and (3) give a model for optimizing resource allocation when preparing for the contamination incident's decontamination stage.

F Masoumi et al. [18] demonstrated the use of an optimization framework based on regret in pollution control throughout many periods in water distribution networks. The paper presented a model for controlling contaminations in water distribution networks that considered uncertainty in pollutant loads. The model employed EPANET software and a genetic algorithm to reduce the time necessary to restore the network to normal, the quantity of contaminants, and the quantity of contaminated nodes. By decreasing Regret, the model incorporated uncertainty. Three types of management tools are used: rapid shutting valves, discharge hydrants, and boosting pumps. In dynamic settings, the suggested model successfully eliminated hazardous pollutants and provided appropriate management solutions.

L Grbčić et al. [19] developed Machine Learning and Simulation-Optimization Coupling for Detecting Pollution Sources in Water Distribution Networks. Two frameworks were constructed, using the Random Forest algorithm for categorization and either the stochastic fireworks optimization algorithm or MADS for optimization. Both frameworks were tested on networks of various sizes and sensor readings and proved to be successful in establishing the contamination source, start and finish timings, and concentration. The second framework performed very well on a network with hazy sensor readings.

MA Khaksar et al. [20] proposed a revolutionary dynamic hydrant flushing approach to get rid of the amount of injected contaminants as soon as the sensors picked it up. To establish the hydrant flushing methods, the linked EPANET-IHS simulation-optimization model was created. This increases the efficacy of the flushing by enabling water utility management to use the hydrants immediately following the sensor warning without the need for further investigations. The recommended hydrant flushing procedures were dependable to remove any injected contaminated mass since created after analyzing hundreds of contaminant injection situations (occurrences of contamination). Moreover, the operation of hydrant sets was dynamically verified by hydrants being closed and opened during the whole hydrant flushing time, allowing the model to flush up to more than 20 %. The medium-sized WDS of Mesopolis City was used to test the approach.

OS Adedoja et al. [21] created an algorithm for predicting the sources of contamination in a network of water distribution. The transient and steady state circumstances were the two different kinds of WDN analysis problems. This investigation considered a steady-state scenario to identify any potential ongoing contamination in a WDN. The superposition technique was used to construct a model that embedded and linked the distribution of contaminants to a set of supplied data, and an algorithm for solving it was developed to determine the origins of contamination. 1st case: 0.99894, 2nd case: 0.99937, and 3rd case: 0.99974 were the estimated results for the corresponding coefficient of determination, while 1st case: 0.000364, 2nd case: 0.000351, and 3rd case: 0.000299 were the estimated results for the corresponding root mean square, respectively, for a noise level of 5 %. Also found were the same characteristics with a noise level of ten percent. The outcomes attested to the viability of the unique strategy that was suggested and may be used with a bigger water distribution system.

et al. [22] created the best emergency response methods using MO (multi-objective) particle swarm optimization. They also estimated the danger of pollutants getting into the network. Three primary goals make up the issue: reducing the quantity of operational interventions, contaminated nodes, and exposed people. The locations of open hydrants and closed valves were picked as the decision factors. An actual network and a benchmark were used to show how the suggested strategy works.

### 1.2 Research gap

This study is closely related to the mentioned works on consequence management and pollution control within urban water distribution networks. However, there are several gaps in the existing literature that our study aims to address.

Lack of research on consequence management: There is a scarcity of research specifically focused on the management of consequences in urban water networks after vandalism and pollution incidents. The present study endeavors to address this research gap by introducing a novel approach that integrates simulation and optimization techniques to effectively manage reactive activities.

Limited consideration of optimal reactive activities: Existing research often neglects to sufficiently tackle the strategic selection and optimization of reactive measures, such as the strategic placement of shut-off valves and hydrants, to effectively mitigate the spread of pollutants and facilitate the sustainable reuse of water within the network. This study endeavors to address the aforementioned research gap by incorporating the multi-objective modified seagull algorithm into the process of identifying the most suitable locations for these measures. Exploiting the archive capacity to speed up the process of extracting these ideal activities has received less attention.

There is a lack of adequate integration between simulation and optimization models in numerous research since they tend to utilize either simulation or optimization models in isolation when addressing consequence management. The suggested methodology involves the integration of the EPANET simulation model with the MOMSOA (multi-objective modified seagull) optimization model, facilitating a thorough examination and optimization of reactive measures within the water network.

### 1.3 Novelty

Integration of EPANET software and the multi-objective modified seagull algorithm (MOMSOA): This research combines the widely-used EPANET software, a powerful tool for water distribution system analysis, with MOMSOA, which introduces an external archiving mechanism. This integration provides a novel framework for managing consequences in urban water networks.

The optimization of shut-off valves and hydrants warrants consideration. Prior research has predominantly concentrated on detection methodologies and monitoring systems. However, this study introduces a novel perspective by emphasizing the optimization of the placement of shut-off valves and hydrants. The identification of optimal locations is predicated on the objective of containing pollutants and expediting their removal from the network.

The study applies a real-world case study to exemplify the proposed approach. Specifically, Example 3 of the EPANET software is utilized, which represents a realistic urban water network comprising various components, including pipes, nodes, tanks, pumps, and reservoirs. Through the application of the integrated simulation-optimization method to this case study, the research showcases the practical feasibility and effectiveness of this approach for consequence management in practical scenarios.

Overall, the combination of the EPANET software, the MOMSOA algorithm, and the integration of shut-off valves and hydrant optimization in a real-world case study provides new insights into impact management in urban water networks and reduces time Optimum activities are extracted, which have been given less attention. In addition, in this study, a comparative analysis is made between the MOMSOA approach and other optimization approaches including MOSOA, MOMSA, MOPSO, and NSGA-II. This analysis shows the superiority of the MOMSOA approach in terms of reactive activities (cost) and consumption pollution volume.

## 2 Materials and methods

**Fig. 1:**
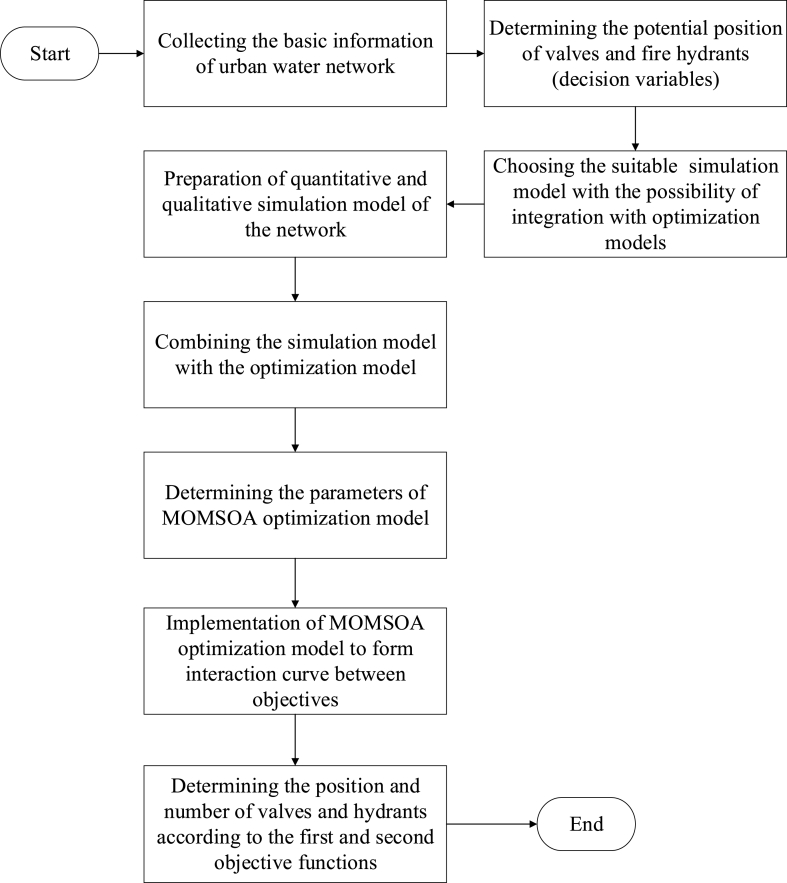
The structure of the suggested method in consequence management.

In this research, EPANET software and its library file in MATLAB program are used as quantitative and qualitative simulation models, multi-objective modified seagull algorithm based on the archive as an optimization model, and example number 3 of EPANET software as a case study. Fig. 1 shows the general structure of the proposed approach in consequence management.

To retrieve the urban water network after carrying out vandalism operations and pollution incidents (consequence management), it is necessary to take appropriate reactive measures to enable the reuse of the system for consumers. The simulation-optimization method in choosing the optimal reactive activities is one of the most widely used approaches in the management of consequences in urban water networks. In this research, the optimal reactive activities of shut-off valves and hydrants are chosen from potential locations to isolate pollution (intercept its spread) and discharge it from the network. As shown in Fig. 1, the proposed approach to managing the consequence is a combination of the EPANET simulation model and the MOMSOA optimization model. In this approach, to Fig. 1, the network's required information, including network topology, consumption pattern, and potential points of shut-off valves, and hydrants are identified. Subsequently, specific polluting features are introduced and entered into the simulation model. In the following, the EPANET simulation model is prepared for hydraulic analysis of the network during consequence management. The parameters of the MOMSOA optimization model are specified and after running the integrated simulation-optimization model, the results are presented as interaction curves between different objectives. The decision variables in this issue are the optimal position of shut-off valves to contain pollutants and keep them from spreading, as well as the optimal position of hydrant valves to discharge pollution from the network.

Using the optimal interaction curve obtained between different objectives, the appropriate number of reactive activities can be determined according to the desired values of the decision-makers from the point of view of the amount of pollution mass consumed after the start of consequence management.

### 2.1 Objective functions

In this research, the objectives used include (a) minimizing the number of reactive activities and (b) minimizing the mass of pollution used up, which are described below.

a) Minimize the number of reactive activities (X1)

The first objective function, X1, according to equation (1), is the total number of reactive activities that be accomplished after the network is contaminated to isolate and drain the contaminated water from the system.

(1) $$F1=\sum_{i=1}^{V}{VS}_{i}+\sum_{j=1}^{H}{HY}_{j}$$

where i specifies the counter of the shut-off valve, ${VS}_{i}$ represents the $i^{th}$ shut-off valve, V indicates the total number of shut-off valves, j illustrates the counter of fire hydrant, ${HY}_{j}$ represents the $j^{th}$ fire hydrant, H signifies the entire quantity of hydrant in the network. The value of ${VS}_{i}$ and ${HY}_{j}$ can be zero or one. If ${VS}_{i}=1$, this means that the operation mode of that valve during the consequence management period helps to isolate a part of the network; Otherwise, it remains in the same normal state (open). Similarly, for ${HY}_{j}$, if its value is equal to one, the operation mode of that hydrant valve in the network is modified to discharge pollution from the network; Otherwise, it remains in the same initial state (closed).

Considering the number of reactive activities as an objective function facilitates a systematic evaluation and optimization of consequence management actions, ensuring an effective and efficient response to water contamination incidents.

b) Minimizing the mass of pollution consumed (X2)

The second objective function, X2, according to equation (2), the amount of pollution consumed by consumers at each node over time in the research is calculated.

(2) $$F2=\sum_{f=1}^{N}\sum_{t=td}^{EPS}C_{ft}\times V_{ft}$$

where, X2 represents the amount of pollution consumed by consumers, which is the objective function being minimized in the research, $\sum_{f=1}^{N}$ represents all the nodes (f) in the network (N). The variable td represents the time passed after the detection of pollution in the network until the start of the simulation, and EPS represents the end of the simulation time. $C_{ft}$ denotes pollutant concentration at node f in time t, and $V_{ft}$ illustrates the volume of polluted water consumed at node f in time t.

Therefore, Equation (2) presents a comprehensive depiction of the collective pollution intake by all consumers in the network, taking into account the temporal fluctuations in pollutant concentration and water consumption rate at each node. This aforementioned equation holds considerable significance in evaluating the effectiveness of diverse pollution mitigation techniques, as well as in optimizing the design and operation of water distribution systems.

In equation (2), $C_{ft}$ is utilized in the computation of the quantity of pollution that is absorbed by consumers. A modeling technique is employed to determine the concentration of pollutants at a certain point in time.

To simulate water quality, the EPANET software was employed, which is a widely utilized software for modeling water distribution systems. This software employs mathematical models that are specifically designed to simulate water quality in distribution systems. These models take into account various factors such as hydraulic flow, reaction kinetics, mixing, and other water quality parameters, and are capable of estimating pollutant concentration at different nodes over time.

According to equation (2), there is a time step involved. The variable "t" represents the temporal increment or temporal index within the summation ∑t=td to EPS. In this study, the time step is utilized as a mechanism to emulate the dynamic features of the water distribution system and observe fluctuations in pollutant concentrations over the temporal domain. The selection of the time step duration is dependent on the research requirements and the intrinsic attributes of the system being modeled. In the context of this particular scenario, a time interval of 1 h is considered suitable.

### 2.2 EPANET software

Urban water network simulation models are models that simulate the hydraulic and qualitative behavior of water in pipes under pressure in different time frames. The general form of all common hydraulic and water quality simulation models is mostly the same and all of them have a set of equations that must be solved according to the initial conditions, boundary conditions, and hydraulic conditions in different time steps. EPANET is one of the famous models for quantitative and qualitative simulation of water in urban WDN (water distribution networks). The US Environmental Protection Agency's Department of Water Resources and Research Laboratory Studies developed this program, which is extensively used across the world. The hydraulic and qualitative modeling of the network in this study is carried out using EPANET 2.0. The EPANET Toolkit, a side package of this model, has been utilized in conjunction with the MATLAB (version R2018b) coding language due to the requirement for repeated executions of this model in conjunction with the model of optimization. The following is an exhaustive exposition of EPANET, encompassing its various attributes and functionalities:

The EPANET software offers users the ability to simulate complex water distribution networks by specifying various elements, including nodes, pipes, tanks, pumps, valves, and other relevant components. Nodes are used to indicate junctions or demand locations within a system, while pipes are utilized to establish connections between these nodes, representing the distribution pipelines.

The hydraulic analysis process entails the utilization of software to calculate the water flow within a network, taking into account several factors such as pipe diameter, length, roughness, elevation, and nodal demands. EPANET employs the fundamental principles of hydraulics and equations that govern the dynamics of fluid flow to determine various hydraulic attributes, including pressures, velocities, head losses, and other relevant properties within the network.

The EPANET software enables the simulation of water quality by providing a platform for modeling contaminant transport and water age within a designated system. This simulation incorporates a range of parameters, such as initial concentrations, reaction rates, decay coefficients, and source strengths. Through the consideration of these factors, EPANET is capable of predicting the spatial and temporal distribution of water quality properties.

EPANET enables the simulation and analysis of pumping and control systems through its capacity to model diverse components, including pumps, valves, and control devices such as pressure-reducing valves and flow controllers. The software emulates the operation and effects of pumps on system performance, thereby facilitating the optimization of pump scheduling, setpoints, and control techniques to achieve the desired hydraulic conditions.

EPANET facilitates the evaluation of scenarios by providing users with the ability to adjust various elements of the network, including network components, operational settings, and demand patterns. This feature enables the comparison of multiple scenarios, thereby facilitating the evaluation of the effects of system adjustments, the formulation of expansion plans, and the assessment of the efficacy of control mechanisms.

The software provides a Graphical User Interface (GUI) that simplifies user interaction through an aesthetically pleasing and user-friendly interface. This GUI empowers users to effortlessly model networks, perform modifications, and straightforwardly visualize outcomes. Users can expeditiously design, modify, and assess networks, as well as visualize hydraulic and water quality results using diverse graphical and tabular displays.

In the study's analysis of the network's hydraulics, a supply-and-demand methodology is adopted. This methodology aims to ensure that the water demand at each node in the network is sufficiently met. It takes into account the varying demands placed on the network at different locations and times. By simulating how water is supplied to meet these demands, the study gains insights into how the network operates under different scenarios and conditions. To understand the flow characteristics of water in the network, the study evaluates the relationship between water head loss and the network using Hazen-Williams analysis. Hazen-Williams formula is a widely used empirical equation that estimates head loss based on factors such as pipe length, diameter, roughness, and flow velocity. By applying the Hazen-Williams analysis, the study assesses how head loss affects the flow of water within the network and evaluates the efficiency of the system. To capture the dynamics of the network over time, the simulation considers a 24-h time frame. This allows for observations and analysis of water flow patterns and characteristics throughout a typical day. The hydraulic calculations are performed at hourly intervals, providing a detailed understanding of how the network's hydraulic behavior evolves. Additionally, qualitative analysis is conducted at 5-min intervals to capture nuances and variations in water quality parameters. By adopting this time-based approach, the study gains insights into how the network performs under different demand patterns, hydraulic conditions, and water quality variations throughout the day. This information can be valuable for optimizing the network's design, operation, and maintenance, ensuring efficient and reliable water supply to consumers.

An assumption is made that the chemical substance used in the simulation remains stable without undergoing any chemical reactions with water or the pipe walls. This assumption allows the focus to be placed on analyzing the hydraulic behavior of the water network, without considering additional complexities introduced by chemical reactions. To ensure accurate and reliable simulation results, several settings play a crucial role in the functioning of the simulation process. These settings are typically defined within the software used for the simulation, such as EPANET. Let's take a closer look at the specific settings mentioned:

CHECKFREQ: This setting determines the frequency at which the hydraulic equations governing the flow of water in the network are checked for convergence. A default value of 2 means that the convergence check is performed every 2 iterations of the simulation. By monitoring convergence, the simulation can ensure that accurate and stable results are obtained.

ACCURACY: The ACCURACY setting defines the level of accuracy in the solution obtained for the hydraulic equations. It represents a tolerance limit for hydraulic calculations. A smaller value, such as 0.100, indicates a higher level of accuracy and precision in the simulation results.

MAXCHECK: This setting determines the maximum number of consecutive times that hydraulic convergence can fail before the simulation is terminated. If convergence fails repeatedly, it suggests that the network configuration or input parameters may need adjustment. The default value of 10 provides a balance between detecting convergence issues and allowing for minor fluctuations during the simulation.

DAMPLIMIT: DAMPLIMIT is a setting that controls the degree of damping or smoothing applied to stabilize the hydraulic calculations. A value of zero implies no damping is applied. Damping helps to prevent oscillations or instability in the simulation results, especially when there are abrupt changes in demand or hydraulic conditions.

These settings [23] are carefully chosen to strike a balance between accuracy, stability, and computational efficiency in the simulation process. They contribute to obtaining reliable results that reflect the hydraulic behavior of the water network under investigation.

### 2.3 Modified Seagull optimizer algorithm

The modified seagull optimization method was the algorithm employed in this study [24]. The Seagull Optimizer (SO) method is further described in the subsequent paragraphs.

#### 2.3.1 Immigration (exploration)

An immigration procedure imitates the movements of a seagull swarm into the positions. This approach executes algorithm exploration. Three criteria should be wanted in immigration, namely.

I) Collisions are avoided by periodically updating the swarm's position by an additional parameter, V, as shown in equation (3).

$${\vec{P}}_{N}=V\times {\vec{P}}_{c}\left(i\right)\text{,}$$

(3) i=0,1,2,...,Max(i)

where ${\vec{P}}_{c}\left(i\right)$ represents the position of the candidate in the current iteration, ${\vec{P}}_{N}$ is the position that prevents it from interacting with the different search candidates, and V describes the motion behavior of the search candidate in the solution space and is represented as follows [equation (4)].

(4) $$V=f_{c}-\left(i\times \left(\frac{f_{c}}{\mathrm{Max}\;\left(i\right)}\right)\right)$$

where V's frequency management between 0 and fc is indicated by the symbol $f_{c}$ and i denotes the iteration.

Equation (4) delineates how the variable V undergoes alterations during successive rounds. As the iterations proceed, the value of V steadily lowers, enabling the search candidates to conduct a more thorough exploration of the solution space during the first phases. As the algorithm undergoes further development, the level of investigation diminishes, leading the candidates to prioritize the exploitation of local regions that are anticipated to have viable answers.

The immigration process in the Seagull Optimization Algorithm employs equations (3), (4) to ascertain the updated locations for the seagull candidates. The utilization of these equations guarantees that the candidates effectively evade collisions and demonstrate deliberate exploratory behavior during the entirety of the optimization procedure. The method achieves a trade-off between exploration and exploitation by modifying the value of V by the iteration count. As a result, it can conduct a search that is both efficient and successful in finding solutions that are optimal or close to optimal.

II) While preventing collisions, the candidates seek to go towards the optimal option (best solution) by trying to exploit the information of their neighbors [equation (5)].

(5) $${\vec{d}}_{e}=K\times \left({\vec{P}}_{b}\left(i\right)-{\vec{P}}_{c}\left(i\right)\right)$$

where vector ${\vec{d}}_{e}$ describes the relative placements of each candidate (${\vec{P}}_{c}\left(i\right)$) to the best-matched candidate (${\vec{P}}_{b}\left(i\right)$). The value of the random-chosen coefficient K controls the ratio of exploitation to exploration. The equation illustrated below is used to get K [equation (6)].

(6) $$K=2\times V^{2}\times R$$

where a random number in the interval of 0–1 is assigned to R.

The calculation of K in equation (6) incorporates both the motion behavior parameter V and a random component R. The value of K influences how much the candidate seagulls exploit the information from the best-matched candidate compared to exploring the solution space further. By adjusting the value of K, the Seagull Optimization Algorithm can strike a balance between exploitation and exploration. A higher value of K prioritizes exploitation, enabling candidates to move towards the best solution more intensively. On the other hand, a lower value of K promotes exploration, allowing candidates to search for alternative solutions in unexplored regions of the solution space.

III) The search agents progressively approach the best answer, and then they adjust their location based on the best solution found as follows [equation (7)]:

(7) $${\vec{D}}_{e}=\left|{\vec{P}}_{N}-{\vec{d}}_{e}\right|$$

where the difference between the seagulls and the ideal solution is represented by ${\vec{D}}_{e}$.

Equation (7) quantifies the distance between the seagull candidates and the ideal solution or the best answer found so far. By calculating this difference, the algorithm obtains information about how far each candidate is from the optimal solution. Based on this information, the candidates adjust their locations to gradually approach the best solution. The adjustment of the seagull candidates' locations is performed by minimizing the value of ${\vec{D}}_{e}$. As the algorithm progresses, the positions of the candidates are updated iteratively to minimize the difference between their current locations and the ideal solution. By continuously adjusting the locations of the search agents based on the best solution found, the Seagull Optimization Algorithm aims to converge toward the optimal or near-optimal solution gradually.

Equation (7) calculates the difference vector between the seagull candidates' positions after immigration and the relative placements vector towards the best-matched candidate. This difference vector quantifies the distance between the candidates and the ideal solution, allowing for iterative adjustment of their locations to approach the best answer.

#### 2.3.2 Attacking (exploitation)

Seagulls can regularly change the velocity and angle of their flight while migrating, and they can also keep their position in the flight by using their mass and feathers. The algorithm's exploitation phase is represented by this process. Throughout the attack, seagulls travel in a spiral pattern in the air along the x, y, and z axes [equations (8), (9), (10)]:

(8) $$\hat{x}=r\times \cos\left(t\right)$$

(9) $$\hat{y}=r\times \sin\left(t\right)$$

(10) $$\hat{z}=r\times t$$

where the value of "r" is the spiral turn radius and is described by equation (11), while "t" is a random number between 0 and 2.

(11) $$r=\alpha \times e^{\beta t}$$

The spiral's form is signified by the symbols *α* and *β*, while "e" stands for the natural logarithm's base. To update the seagull location, use equation (12).

(12) $${\vec{P}}_{c}\left(i\right)=\left({\vec{D}}_{e}\times \hat{x}\times \hat{y}\times \hat{z}\right)+{\vec{P}}_{b}\left(i\right)$$

where the ideal results are stored in vector $P_{c}\left(i\right)$.

Premature convergence and rate of convergence are the two main problems with SO. To fix these flaws, a solution is provided below.

#### 2.3.3 Modified SOA (MSOA)

The section discusses the modified SOA, which makes use of Lévy flight to increase the SOA's efficacy. This strategy's main goal is to get around the premature convergence problem, which is a significant SOA limitation. A new random walk feature in Lévy Flight makes it easier to better handle local searches (as described by Ref. [25]). Listed below is how this process works [equations (13), (14), (15)]:

(13) $$Le\left(w\right)\approx w^{-1-\tau }$$

(14) $$w=\frac{V}{{\left|K\right|}^{1/\tau }}$$

(15) $$\sigma^{2}={\left\{\frac{\Gamma \left(1+\tau \right)}{\tau \Gamma \left(\left(1+\tau \right)/2\right)}\frac{\sin\;\left(\pi \tau /2\right)}{2^{\left(1+\tau \right)/2}}\right\}}^{\frac{2}{\tau }}$$

where τ indicates the Levy index, and between 0 and 2 (τ=3/2 [26]), $V∼N\left(0,\sigma^{2}\right)$ and $K∼N\left(0,\sigma^{2}\right)$, Γ(.) specifies the Gamma function, *w* displays the step size, and V/K ∼ N(0, σ 2) implies that the specimens were produced from a Gaussian distribution with a mean of 0 and a variance of $\sigma^{2}$, respectively.

The newly enhanced component for updating the SOA solution is based on the aforementioned approach and is as follows [equation (16)]:

(16) $${\vec{D}}_{el}={\vec{D}}_{e}+\left|{\vec{P}}_{N}+{\vec{d}}_{e}\right|\times \text{Le}\left(\delta \right)$$

where the search candidate's new place is represented by the symbol " ${\vec{D}}_{el}$".

The most appropriate candidates are maintained by applying equation (17) to arrive at the optimum solution by candidates:

(17) $${\vec{D}}_{el}=\left\{ \begin{array}{c} {\vec{D}}_{el}\;F\left({\vec{D}}_{el}\right)>F\left({\vec{D}}_{e}\right)\\ {\vec{D}}_{e\;}otherwise \end{array}\right.$$

**Fig. 2:**
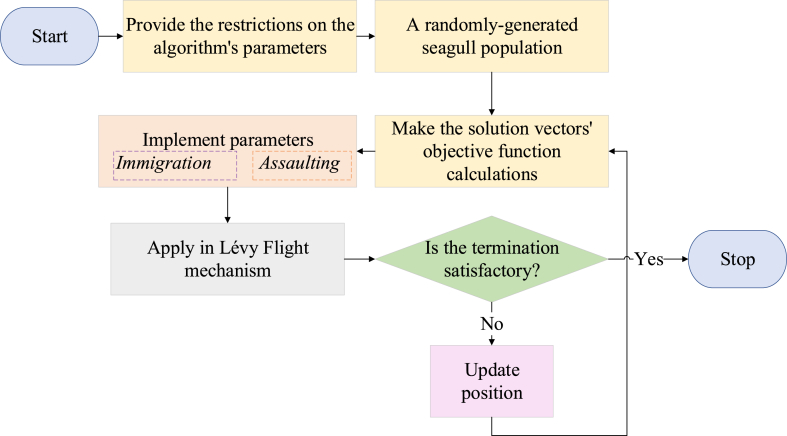
The suggested MSOA flowchart schematic.

This approach is used in place of the traditional strategies to prevent becoming caught in the local minima. The modified seagull optimization algorithm's structure is shown in the following figure (Fig. 2).

#### 2.3.4 Modified multi-objective seagull optimization algorithm

The present study integrates the MSOA [27] approach with a multi-objective optimization framework to introduce a novel technique referred to as multi-objective MSOA (MOMSOA).

In contrast to a single-objective optimization technique, the multi-objective optimization issue involves the consideration of many sub-objectives that may conflict with one another. Consequently, conventional comparison operators prove inadequate in the pursuit of identifying the most effective option. In the context of multi-objective optimization issues, it is customary to depict feasible answers as a collection of Pareto optimal solutions, as opposed to a solitary solution. For a solution to be considered Pareto optimum, it must adhere to the requirement of either dominating all other solutions concerning at least one objective or not being dominated by any other solution. The Pareto solutions are saved within an archive that has a predetermined maximum capacity. If the quantity of Pareto solutions is above the predetermined capacity, the archive will remove the Pareto solutions with the lowest level of crowding.

The roulette approach is utilized within the framework of the Multi-Objective Modified Seagull Optimization algorithm (MOMSOA) to select an individual from the archive as the seagull that represents the global optimum. The present selection is derived from the subsequent formula [equations (18), (19)]:

(18) $$p\left(x_{i}\right)=\frac{f\left(x_{i}\right)}{\sum_{j=1}^{L}f\left(x_{i}\right)}$$

(19) $$q\left(x_{i}\right)=\sum_{j=1}^{i}p\left(x_{j}\right)$$

Where, $f\left(x_{i}\right)$ denotes the fitness of an individual $x_{i}$, $p\left(x_{j}\right)$ represents the probability of selecting that specific individual, $q\left(x_{i}\right)$ represents the cumulative probability of selection, and L corresponds to the population size of seagulls.

The Multi-objective modified seagull optimization introduces an external archiving mechanism into MSOA to simultaneously optimize different objective functions.

• External archive method

The solutions discovered while executing MSOA are saved in the MOMSOA (multi-objective modified seagull optimization algorithm), which incorporates the external archive into MSOA. The MOMSOA employs an external archive for storing the finest solutions found during the search for multi-objective optimization algorithms [28]. The external archive is a secondary data structure that is kept in addition to the algorithm's population of candidate solutions. The external archive's primary goal is to prevent the loss of good solutions discovered during the search process. When a solution is discovered that is better than any of the existing solutions in the archive, it is added to the archive. If the archive is full, the new solution takes the place of the worst solution in the archive.

In this research, initially, an archive with a specific limit is established to accommodate diverse types of seagulls (solutions). By the modified multi-objective seagull algorithm, every novel solution will be incorporated into the archive during each iteration, provided that the archive's capacity limit has not been met and the solution is not already present in the archive. In the condition that the maximum capacity has already been reached or the solution is already contained in the archive, the preceding solution will be retained in the archive. Additionally, if the archive has reached its maximum limit and no novel solution is present, the solution with the worst value for the objective function will be deleted. As a result, the archive provides different and appropriate solutions in terms of the objective function.

### 2.4 Network specifications

**Fig. 3:**
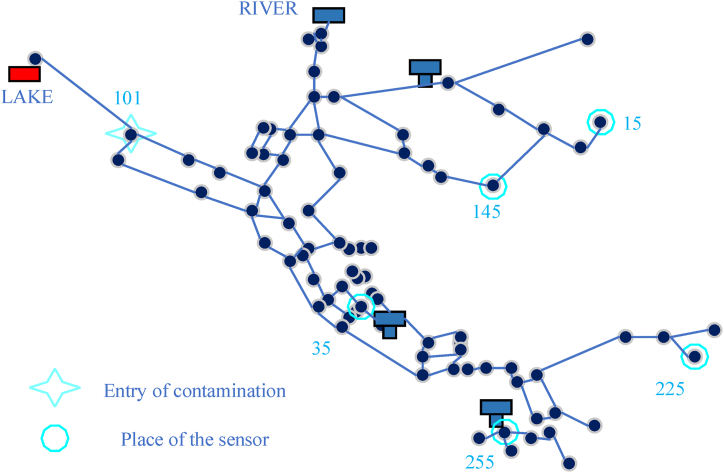
Network specifications, Example 3 EPANET Software.

Example 3 in EPANET software has been used in several investigations conducted in this field. 117 pipes, 92 nodes, 3 tanks for storing, 2 pumps, and 2 reservoirs, including a lake and a river, make up this network (Fig. 3). In this specific water distribution network, the study assumes that contamination is intentionally introduced from node 101 at 9:00 a.m. The contamination continued to spread throughout the network until 7:00 a.m. the next day. This assumption allows for the analysis and evaluation of the network's response to a deliberate pollution event. To optimize the likelihood of detecting pollution and identifying the source, the Steinfeld and Salomons technique is employed. This technique focuses on strategically placing sensors within the network. In this case, five sensors are placed at nodes 15, 35, 145, 225, and 255. By strategically positioning the sensors, the study aims to enhance the chances of promptly detecting and locating the pollution source [29].

Using the Stefeld and Salomons technique, it is determined that the sensor located at node 35 successfully detects pollution at 10:45 a.m. This detection triggers further actions to locate the exact source of the pollutants and initiate reactive measures. It is assumed that it takes approximately 1 h to complete this process. From 12:00 a.m. onwards, reactive activities are implemented based on the detected pollution. These activities involve taking appropriate actions such as isolating contaminated sections of the network or adjusting the operations of pumps and reservoirs. The reactive actions continue until the simulation time is over at 12:00 p.m.

By employing this simulation scenario, the study aims to evaluate the effectiveness of pollution detection techniques, assess the network's ability to respond to pollution incidents and analyze the efficiency of reactive actions in a specific time frame.

## 3 Results

The simulation-optimization technique has been utilized in this study to identify the optimal strategy for managing consequences in urban water distribution networks following a pollution incident. One hour is allocated once the contamination is discovered for planning the best course of action and sending out reaction teams. Therefore, optimal response activities include closing and opening hydrants starting at 12 a.m. This method assumes that the optimal response action remains constant over the duration of the consequence management period. Table 1 lists the locations of the 19 shut-off valves and 30 hydrant valves that are considered to isolate the network and discharge pollutants, respectively.

**Table 1:** Position of Potential valves and Hydrants in a scenario of consequence management.

The position of the shut-off valves in the pipes	The position of the potential hydrant valves
108, 112, 117, 124, 156, 174, 176, 178, 205, 216, 222, 230, 232, 237, 269, 301, 309, 311, 317	45, 55, 62, 70, 121, 130, 165, 170, 174, 180, 182, 184, 185, 188, 196, 205, 207, 209, 242, 250, 256, 260, 262, 264, 266, 268, 270, 272, 274, 276

Therefore, the MOMSOA multi-objective optimization model contains variables for binary decisions, and the whole quantity of decision variables is equivalent to the entire number of shut-off valves and potential hydrant valves in the network.

**Fig. 4:**
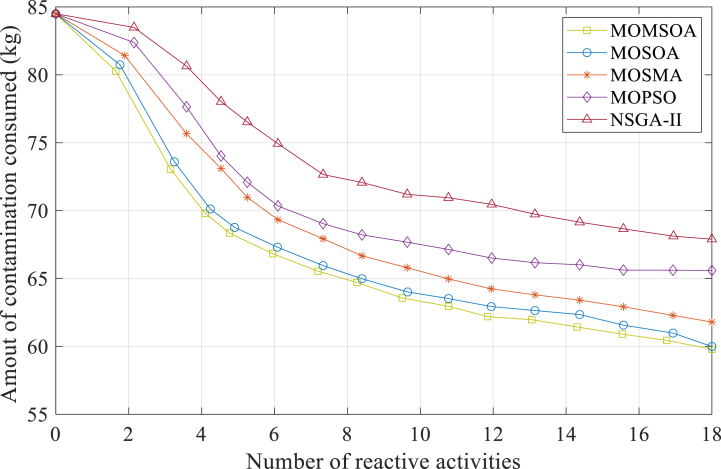
The curve of interaction between the several consequence management objective.

The optimal number of reactive activities (F1) can be achieved by the intended quantity to reduce the mass of contamination consumed (F2) by applying the acquired interaction curve between the objectives. A maximum of 18 response teams have been taken into account for consequence management in this study. Before beginning the optimization method, the optimization model's parameters are specified. Sensitivity analysis is utilized to specify the optimal value for each of these factors. The MOMSOA model's population consists of 60 seagulls, and its execution will terminate when it achieves the stopping conditions, which include the maximum number of iterations and the amount of improvement in the objective function. To compare the proposed method with other algorithms, Fig. 4 shows the interaction curve between the number of reactive activities and the quantity of consumed pollutant mass. Each graph is related to the implementation of a multi-objective algorithm. The comparative methods include the multi-objective optimization of the Seagull optimization algorithm (MOSOA), NSGA-II [30], the multi-objective optimization of the particle swarm (MOPSO) [31], and the multi-objective slime mould algorithm (MOSMA) [32].

About the convergence speed of the algorithms. MOMSOA demonstrates remarkable efficiency in terms of convergence by achieving the desired interaction curve between the number of reactive activities and the quantity of consumed pollutant mass within approximately 80 min, as illustrated in Fig. 4. By reducing the time required for extracting optimal activities, MOMSOA enables swift implementation of reactive actions, thereby enhancing the overall effectiveness of pollution control strategies. Then, the quality of the solutions obtained by each algorithm are examined. Our proposed MOMSOA consistently yields high-quality solutions, as evidenced by the achieved reduction in pollution consumption. Specifically, after applying 18 reactive actions, MOMSOA successfully reduces the mass of contaminations consumed to 59.8 kg, outperforming MOSOA, MOSMA, MOPSO, and NSGA-II which achieve reductions of 60, 61.8, 65.6, and 67.9 kg, respectively. This demonstrates the superior performance of MOMSOA in minimizing pollution and ensuring efficient consequence management.

The advantages offered by our proposed MOMSOA approach are explained as follows. Unlike traditional methods, MOMSOA incorporates an archive-based strategy that allows for the avoidance of simulating similar seagull locations during the optimization phase. By specifying an archive with a specific capacity, duplicated seagull positions are removed, resulting in a more streamlined and focused optimization process. This innovative approach significantly reduces the extraction time of ideal reactive actions, facilitating prompt decision-making and maximizing pollution control efforts.

Furthermore, MOMSOA tackles the problem of optimizing reactive activities by considering multiple objectives simultaneously. Through the acquired interaction curve between the objectives, MOMSOA ensures the optimal balance between reducing contamination consumption and achieving the desired number of reactive actions. By taking a multi-objective approach, MOMSOA provides decision-makers with a comprehensive understanding of the trade-offs between different aspects of consequence management, enabling informed and efficient decision-making. In conclusion, our proposed MOMSOA algorithm outperforms existing methods in terms of convergence speed and solution quality. With its archive-based strategy and multi-objective optimization framework, MOMSOA offers distinctive advantages, including reduced extraction time for optimal activities and a comprehensive approach to consequence management. These features make MOMSOA a superior choice for addressing the problem of optimizing reactive activities and improving pollution control efforts.

The proposed MOMSOA (multi-objective modified seagull algorithm based on archives) algorithm offers several advantages over the multi-objective seagull algorithm without archives. By incorporating archives, MOMSOA demonstrates improved convergence speed, and enhanced solution quality. MOMSOA utilizes archives to store a diverse set of high-quality solutions obtained during the optimization process. This information guides the search towards promising regions of the solution space, resulting in faster convergence compared to the algorithm without archives. Additionally, the availability of archives allows MOMSOA to maintain a well-distributed set of Pareto-optimal solutions, enabling a comprehensive exploration of the search space and capturing the true trade-offs between conflicting objectives. On the other hand, the absence of archives in the alternative algorithm may limit its ability to identify and preserve a diverse set of non-dominated solutions effectively. Furthermore, the integration of archives in MOMSOA enhances its robustness by preventing premature convergence to suboptimal solutions. By storing non-dominated solutions, the archive ensures that potentially valuable alternatives are not lost during the optimization process. In contrast, the lack of archives in the other algorithm can hinder its ability to recover from local optima and explore new regions effectively.

**Fig. 5:**
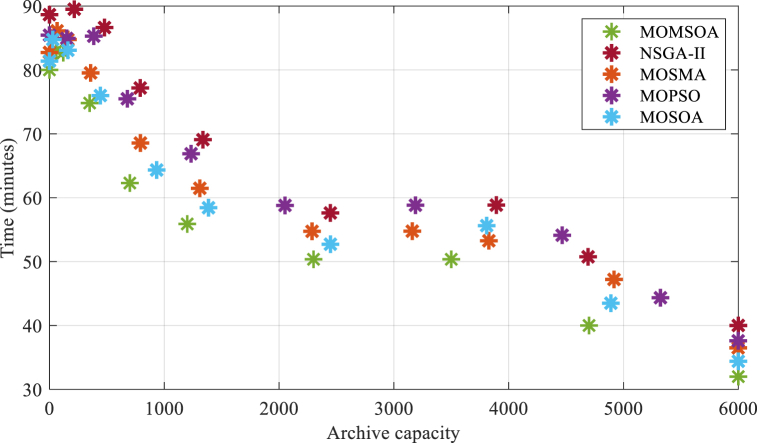
The time needed to extract the interaction curve between the objectives for various archive capacities.

The interaction curve between the objects based on the MOMSOA approach in Fig. 4 takes around 80 min to extract. Reducing this time appears logical in light of the significance of hastening the implementation of reactive actions and its impact on how much pollution individuals consume. The multi-objective modified seagull algorithm based on archives is suggested for this purpose. It is feasible to avoid simulating the similar seagull locations (similar situations of the status of shut-off valves and hydrants) in this technique by specifying an archive with a specific capacity (population) of seagulls throughout the optimization phase. In this method, the implementation of the simulation model is stopped for seagull positions created in the new iteration if they have already been placed in the archive. It is expected that the extraction time of the ideal reactive actions will be shortened by employing this strategy and taking into account the capacity allotted to the archive. Fig. 5 displays the time needed to extract the optimal activities (interaction curve between different objectives). To provide a comprehensive comparative analysis, we compared the performance of our proposed MOMSOA algorithm to NSGA-II, MOPSO, and MOSMA in terms of archive capacity and its impact on the extraction time needed to obtain optimal reactive actions.

As illustrated in Fig. 5, when the archive capacity is set to zero, all algorithms show similar extraction times, ranging from 80 to 90 min. However, as the archive capacity increases, the required extraction time decreases for all algorithms. Interestingly, MOMSOA exhibits a more significant reduction in extraction time compared to other algorithms as the archive capacity increases. For instance, at an archive capacity of 6000, MOMSOA requires only 32 min, which shows a 60 % reduction, while the other algorithms require 36–40 min. This highlights the superior efficiency of MOMSOA in optimizing reactive activities, enabling prompt implementation of pollution control strategies.

Moreover, when a low archive capacity is selected, such as 60 or 120, the extraction time required by all algorithms increases. However, the increase in extraction time is more pronounced for MOMSOA compared to other algorithms. This is because, with a low archive capacity, executing the simulation optimization model spends more time finding similar seagulls/solutions. As a result, the use of low archive capacities is not effective in reducing execution time and may even lead to an increase in the execution time.

In conclusion, the comparison of archive capacity and its impact on extraction time reveals that MOMSOA outperforms other algorithms in terms of efficiency, especially when a high archive capacity is selected. This emphasizes the advantage of using MOMSOA for optimizing reactive activities and enhancing pollution control efforts.

**Fig. 6:**
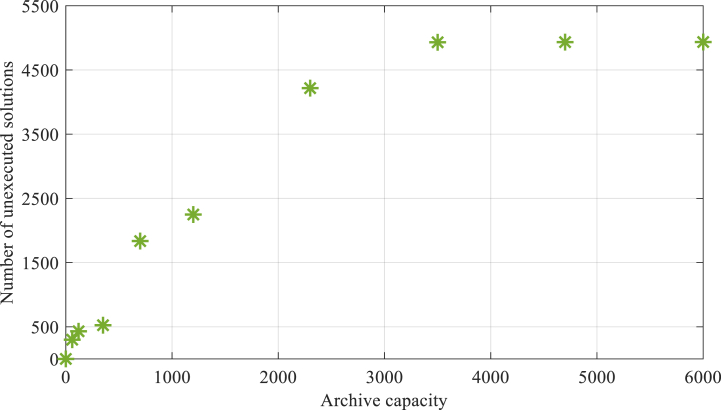
The quantity of unimplemented solutions in the simulation-optimization process for various archive capacities.

In Fig. 6, the number of seagulls (solutions) that were not used in the simulation-optimization process is also shown to give a clearer view of the findings. This information is based on the population in the archive.

As can be observed, the number of seagulls that were excluded from the simulation-optimization process because of their existence in the archive population grows as the archive's capacity increases. It can be seen that by increasing the capacity of the archive from 3500 to 6000, there will not be a significant increase in the number of unexecuted solutions.

## 4 Conclusion

The management of the effects of abrupt contamination in the urban water network has been investigated in this research to extract optimal operation activities using the approach of lowering the optimization time by employing archive capacity. For this purpose, shut-off valves and hydrant valves have been used to isolate pollution and drain it from the network, respectively. This research uses the simulation model of EPANET and the MOMSOA based on archives in the presented hybrid simulation-optimization strategy. Example No. 3 of EPANET software has been used as a case study. This example has been considered in many previous studies of consequence management in urban water networks. In this study, there are two objective functions employed. In this study, the first and second objective functions have been chosen to reduce the number of reactive actions (reducing costs) and the amount of pollution consumed (taking into account public health), respectively. In the first stages of the study, it was discovered that it takes around 80 min to identify the best activities between these aims using a typical MOMSOA and a maximum of 18 activities. This amount of time is seen to be significant for controlling the effects of such occurrences. Using the MOMSOA algorithm based on archives is one of the novelties of this study to shorten this time and enable real-time management of the consequences. In the suggested method, it is feasible to avoid running the simulation model for identical solutions by making use of the archive's capacity, which results in a large decrease in model execution time without a corresponding decrease in model output. In the presented method, the capacity of the archive with the number of zero, 120, 350, 700, 1200, 2300, 3500, 4700, and 6000 seagulls were examined, which also brought good results. For instance, in general, the time needed to extract the interaction curve between objectives drops from 80 to 32 min, which represents a 60 % reduction, as the archive population goes from zero to 6000. This reduction in the management of the effects of the pollution load in the urban water network is significant. In this way, the current research shows that it is feasible to significantly and usefully reduce the time of optimization and management of the consequence in the network in real time by employing an archive. According to the findings, the time needed to extract the ideal activities rises somewhat compared to the base case if a low value is selected for the archive capacity, such as 60 or 120. The reason for this is that if a small value is chosen for the archive capacity, part of the simulation optimization model execution time will be spent on finding seagulls with similar positions. Due to the low capacity of the archive, the increase in the time required to extract similar solutions is greater than the effect of using the archive capacity in reducing the time. It is suggested that the effectiveness of the proposed approach in solving large-scale optimization problems related to urban water networks or quantitative-qualitative exploitation of river-reservoir systems be tested.

## Data availability statement

Research data are not shared.

## CRediT authorship contribution statement

**Qichun Wang:** Writing – review & editing, Methodology, Formal analysis, Data curation, Conceptualization. **Mingxiang Zhang:** Writing – review & editing, Writing – original draft, Validation, Software, Resources, Formal analysis, Conceptualization. **Sama Abdolhosseinzadeh:** Writing – original draft, Investigation, Formal analysis, Conceptualization.

## Declaration of competing interest

The authors declare that they have no known competing financial interests or personal relationships that could have appeared to influence the work reported in this paper.
